# 

**Published:** 1887-11

**Authors:** 


CONTENTS.

Vol. 34 No. 11.

PAGE.

A Remarkable Verification......241
Shakers and Shakerism..........244
Cancer..........................249
What Every Girl Ought to Learn.251
Electrical Mechanism............252
Use of Toilet Soap.............253
Forewarning....................254
Mind Cure......................255
The Olive Tree.................256
The Seybert Commission........257
The Little Maid and the Butterfly.259
Book Notices...................260
Household................ ....261
Miscellaneous..................263

PAGE.

INDEX TO ADVERTISEMENTS OF HALF A PAGE AND OVER.

Premiums of Books.......... I
" " Genuine Silver Plate...................... II
Crystal Pepsin Tablets........ III
Winchester's Hypophosphates. IV
Zylonite...................... IV
Hollow Suppositories.......... V
Celerina...................... VI
Lactopeptine.................. VII
Castoria...................... VII
Crystalline Phosphates........ IX
Goodyear Rubber Co........... IX
Bovinine..................... VIII
Telegraph Medicine Co........ X
Dr. Molesworth............... X
J. B. Watkins' Land Mortgage Co...................... X
Back Numbers as Premiums.. XIII
Lactated Food................ XIV
New York College of Magnetics...........Back Cover

